# Safety and efficacy of a caspofungin-based combination therapy for treatment of proven or probable aspergillosis in pediatric hematological patients

**DOI:** 10.1186/1471-2334-7-28

**Published:** 2007-04-18

**Authors:** Simone Cesaro, Mareva Giacchino, Franco Locatelli, Monica Spiller, Barbara Buldini, Claudia Castellini, Desireè Caselli, Eugenia Giraldi, Fabio Tucci, Gloria Tridello, Mario Renato Rossi, Elio Castagnola

**Affiliations:** 1 Pediatric Hematology Oncology, Department of Pediatrics, University of Padua, Italy; 2 Pediatric Oncology Hematology, Regina Elena Hospital, University of Turin, Italy; 3 Pediatric Hematology Oncology, IRCCS Policlinico San Matteo, University of Pavia, Italy; 4 Pediatric Hematology Oncology, Sant'Orsola Hospital, University of Bologna, Italy; 5 Pediatric Hematology Oncology, G. Di Cristina ARNAS Hospital, Palermo, Italy; 6 Division of Pediatrics, Hospital of Bergamo, Italy; 7 Pediatric Hematology Oncology, Meyer Hospital, University of Florence, Italy; 8 Clinic of Pediatrics, San Gerardo Hospital, Monza, Italy; 9 Division of Pediatric Infectious Disease, "Giannina Gaslini" Institute, Genua, Italy

## Abstract

**Background:**

Fungal infections are diagnosed increasingly often in patients affected by hematological diseases and their mortality has remained high. The recent development of new antifungal drugs gives the clinician the possibility to assess the combination of antifungal drugs with *in-vitro *or in animal-model synergistic effect.

**Methods:**

We analyzed retrospectively the safety and efficacy of caspofungin-based  combination therapy in 40 children and adolescents,  most of them were being treated for a malignant disease, who developed invasive aspergillosis (IA) between November 2002 and November 2005.

**Results:**

Thirteen (32.5%) patients developed IA after hematopoietic stem cell transplantation (HSCT), 13 after primary diagnosis, usually during remission-induction chemotherapy, and 14 after relapse of disease. Severe neutropenia was present in 31 (78%) out of the 40 patients. IA was classified as probable in 20 (50%) and documented in 20 (50%) patients, respectively. A favorable response to antifungal therapy was obtained in 21 patients (53%) and the probability of 100-day survival was 70%. Different, though not significant, 100-day survival was observed according to the timing of diagnosis of IA: 51.9% after HSCT; 71.4% after relapse; and 84.6% after diagnosis of underlying disease, p 0.2. After a median follow-up of 0.7 years, 20 patients are alive (50%). Overall, the combination therapy was well tolerated. In multivariate analysis, the factors that were significantly associated to a better overall survival were favorable response to antifungal therapy, p 0.003, and the timing of IA in the patient course of underlying disease, p 0.04.

**Conclusion:**

This study showed that caspofungin-based combination antifungal therapy is an effective therapeutic option also for pediatric patients with IA. These data need to be confirmed by prospective, controlled studies.

## Background

Fungal infections, especially those caused by *Aspergillus spp*. or by other filamentous fungi, are diagnosed increasingly often in patients affected by hematological diseases.[1,2] Despite the introduction of liposomal and lipid formulations of amphotericin B during the 1990's, infection-related mortality of invasive mycoses has remained high.[3,4] The recent development of new antifungal drugs, such as voriconazole and caspofungin gives the clinician more therapeutic options both for first-line and for salvage therapy of invasive mycoses.[5,6] Notably, caspofungin has a different target of action with respect to the polyenes and triazoles, i.e. it inhibits the synthesis of a component of the fungal cell wall, namely beta-1,3-D-glucan; and data obtained either *in-vitro *or in animal models have shown that the combination of caspofungin with either amphotericin B or voriconazole may exert a synergistic effect. [7-9] On the basis of these premises, several authors have explored the use of caspofungin in combination with either liposomal amphotericin B, itraconazole or voriconazole in patients with invasive mycoses refractory to first-line treatment, with reported response rates ranging between 42 and 60% in the largest series. [10-14] Despite that published pediatric data on the use of caspofungin in combination with other systemic antifungal drugs are limited to single-center experience [15-17], recent multicenter studies showed that, as in adults, this therapeutic strategy is being increasingly adopted by pediatric centers. [18,19]

In this study, we report the data collected among centers belonging to the Italian Association of Pediatric Hematology Oncology (AIEOP) to investigate the safety and efficacy of caspofungin in combination with other systemic antifungal drugs.

## Methods

From January 2002 to December 2003, the AIEOP centres performed a prospective surveillance study aimed at assessing the incidence and outcome of invasive fungal infection in children and adolescents affected by hematological and oncological diseases. [19] During the first year of study, it was noted that caspofungin was often used in combination with other antifungal drugs both as front-line and rescue treatment for invasive aspergillosis (IA). Therefore, a registry was established starting in November 2002 in order to collect prospectively the data on the antifungal combination therapy for IA in children. Each investigator sent to the principal investigator (S.C.) the main clinical and microbiological data of the patients developing IA and treated within 30 days from diagnosis with combination antifungal therapy. Informed consent was obtained from parents or patient's legal representatives. Recruitment of patients was closed on November 2005 and follow-up data are as 31^st ^January 2006.

The eligibility criteria were as follows: pediatric hematological or oncological patients treated with a caspofungin-based combination antifungal therapy for proven or probable IA diagnosed whilst on chemotherapy or after hematopoietic stem cell transplantation (HSCT). Since this was a retrospective study, the main objectives of the study were the definition of a favorable response rate, 100-day survival and overall survival (OS) of patients treated with a caspofungin-based combination therapy, as well as the safety and toxicity of the combination regimen.

*Management of febrile patients*: neutropenic and HSCT patients were nursed in reverse-isolation or high-efficiency particulate-filtered air (HEPA) rooms, respectively. Published recommendations were used for diagnosis and treatment of febrile episodes [20,21], i.e a) ensuring prompt clinical and microbiological evaluation of patients with a search for clinical foci of infection by physical examination, chest X-ray, abdominal ultrasound (if appropriate), cultures of peripheral and central venous catheter (CVC) blood, and, if indicated, mouth and CVC exit-site swabs, stool and urine cultures; b) intravenous administration of broad-spectrum antibiotic therapy for at least 72–96 hours and, in case of persistence of fever, c) a thorough re-assessment of the patient and introduction of empiric antifungal therapy based on either amphotericin B or a lipid/liposomal derivative.[22,23] In recent years, these guidelines have been implemented in patients considered at higher risk of invasive fungal infection, through the use of chest computed tomography (CT) scanning early in the course of the febrile episode for patients not responding to broad spectrum antibiotic therapy and the determination of serum galactomannan. [24-26] The following groups of patients underwent this more thorough diagnostic work-up at the participating AIEOP centers: patients with acute myeloid leukemia; patients with *de novo *or relapsed acute lymphoblastic leukemia while on induction or re-induction therapy; patients undergoing HSCT; patients with prolonged severe neutropenia or on steroid therapy.

For the purposes of the study, the caspofungin-based combination therapy was considered as introduced early (group A) or late (group B) if started within or after 7 days from diagnosis of IA, respectively.

*Definitions*: according to the timing of diagnosis of IA, we distinguished 3 groups of IA: a) those occurring after HSCT, if the patient have been transplanted before IA and, in case of malignant disease, did not experience a subsequent relapse; b) those diagnosed after relapse, if the patient had relapsed prior to the diagnosis of IA; and c) those developing IA during remission-induction chemotherapy for newly diagnosed malignancy, or in case of non-malignant disease, after diagnosis of underlying disease. Regarding the status of the underlying malignant disease at the time of diagnosis of IA, we distinguished 2 groups of patients: those in complete remission, and those without an adequate control of disease, i.e. other remission status.

Severe neutropenia was defined by an absolute neutrophil count < 0.5 × 10^9^/l. Drug-related side effects, organ toxicity and complications after HSCT were defined according to standard criteria.[27]

Proven and probable IA were defined according to internationally accepted criteria. [28] For the purposes of this study, the caspofungin-based combination therapy was considered as primary therapy if the patients were not receiving any mould-active antifungal drug or were on prophylaxis with fluconazole or itraconazole; and as salvage therapy if the patients were receiving empirically or therapeutically any mould-active antifungal monotherapy, at diagnosis of IA, respectively.

The response to antifungal treatment was defined on the basis of the Denning criteria [29], as follows: complete response (CR) was the resolution of all clinical signs and symptoms attributable to IA, together with complete or very nearly complete radiographic resolution (≥ 90%); partial response (PR) was a major improvement or resolution of the attributable clinical signs and symptoms together with at least a 50% improvement in radiological signs; stable response (SR) was consistent with some but less than 50% radiological improvement; and Failure (F) was progression of, or death from, IA. Favorable (or major) response comprised both CR and PR.

### Statistical analysis

Demographic, clinical and microbiological characteristics of patients and infectious episodes were collected through specific case-report forms filled-in by the investigators; data were stored on an Access 97 data base (Microsoft, Seattle, WA, USA). Analysis used January 31^st ^2006 as the reference date, i.e., the day at which all centers locked data on patient outcomes. Where appropriate, the characteristics of the patients and of infectious episodes were compared using chi-square or Fisher's exact test for categorical variables.

The end points of the study were: rate of favorable response, i.e. complete and partial response; 100-day survival, OS, safety and toxicity of the antifungal combination therapy.

One-hundred-day survival and OS were calculated from the date of diagnosis of invasive fungal infection (IFI) to 100 days after diagnosis of IFI, or to the date of death due to any cause or to the date of last follow-up, respectively, by the Kaplan-Meier method. Differences between patients who received a caspofungin-based combination therapy as first line (group A) or rescue therapy (group B) were compared by the log-rank test.

The following variables were included in the analysis of prognostic factors predicting favorable response, 100-day survival and OS: patient gender (M vs. F); median age at diagnosis of IA; occurrence of IA after diagnosis, after relapse or after HSCT, respectively; presence of severe neutropenia at time of diagnosis of IA; single organ vs multiple organ involvement; type of IA (documented vs. probable); surgical treatment of IA; early introduction of caspofungin, i.e. group A vs. group B; and caspofungin-based combination therapy given as primary vs. salvage treatment. Moreover, the rate of favorable response to antifungal therapy was included in the analysis of prognostic factors for 100-day survival and OS. The variables proving significant in univariate analysis were included in a multivariate analysis: favorable response and 100-day survival were assessed by a stepwise logistical regression analysis, whilst OS was determined by a Cox regression analysis. All reported p values are 2-sided, and a significance level of α = 0.05 was used. The statistical analysis was performed using the SAS statistical program (SAS Institute, Cary, NC, Version 8.2).

## Results

The main demographic and clinical characteristics of the patients are shown in table 1. During the study period, 40 patients, 21 males and 19 females, were recruited, median age at diagnosis being 11.1 years (range 1.2–17.9 years).

**Table 1:** Main demographic and clinical characteristics of patients  included in this study.

	**Group A**	**Group B**	**Total**
**Number of patients**	22	18	40
**Gender**			
Male	14	7	21
Female	8	11	19
**Age at IA diagnosis**			
Median (years)	11.94	8.64	11.05
Range	(1.3–17.2)	(1.18–17.9)	(1.18–17.9)
**Underlying disease**			
ALL-AML	15	13	28
CML	2	0	2
NHL+HD	2	2	4
MDS	2	1	3
Non-malignant diseases	1	2	3
**IA after relapse of underlying disease**	9	5	14
Interval from relapse to IA (days)			
Median	69	105	74
Range	(18–174)	(7–261)	(7–261)
**IA after allogeneicHSCT**	7	6	13
Source of SC			
BM	7	4	11
PB	/	1	1
CB	/	1	1
**Interval from HSCT to IA (days)**			
Median	14	15	14
Range	(4–308)	(0–54)	(0–308)
**IA after diagnosis of underlying disease**	6	7	13
Interval from diagnosis to IA (days)			
Median	115	46	74
Range	(15–318)	(13–76)	(13–318)
Remission status of underlying disease *	21	16	37
Complete remission	6	7	13
Other status	15	9	24

Group A comprises patients given caspofungin-based combination therapy  starting within 7 days from diagnosis of IA;  Group B consists of patients given caspofungin-based combination therapy starting after 7 days from diagnosis of IA.

IA, invasive aspergillosis; ALL, acute lymphoblastic leukemia; AML, acute myeloid leukemia; NHL, non-Hodgkin Lymphoma; HD, Hodgkin Lymphoma;  MDS, myelodysplastic syndrome; HSCT, hematopoietic stem cell transplantation; BM, bone marrow; PB, peripheral blood; CB, cord blood; Other status: refractory or progressive disease; *only for the 37 children with malignant diseases.

Thirteen (32.5%) patients developed IA after allogeneic unrelated (10) or related (3) HSCT. The median time from HSCT to IA was 14 days (range 1–308). In the month before the diagnosis of IA, 10 and 2 patients had been treated with steroids at median dose of 2 mg/kg/day for acute GVHD (grade I-II, 5; grade III-IV, 5) and for extended chronic GVHD, respectively.

In 13 patients (32.5%), IA occurred after a median time of 74 days (range 13–318) from the diagnosis of underlying disease whilst in 14 patients (35%) IA occurred after a median time of 74 days (range 7–261) from relapse.

*Characteristics of episodes of fungal infections*: Table 2 summarizes the main data on episodes of IA. They were classified as probable in 20 (50%) and proven in 20 (50%) patients, respectively. In proven IA, the species identification obtained by culture was as follows: *Aspergillus fumigatus*, 7; *Aspergillus flavus*, 3; in the remaining 10 episodes of proven IA, histopathology on tissue sample showed the presence of filamentous fungi consistent with *Aspergillus spp*.

**Table 2:** Main data on invasive mycosis.

	**Group A**	**Group B**	**Total**
**Type of mycosis**			
Probable	13	7	20
Documented	9	11	20
**Severe neutropenia **at diagnosis of IFI	17	14	31
**Lymphocyte count **at diagnosis of IFI			
Median (× 10^9^/L)	100	200	170
Range	(0–2230)	(0–5710)	(0–5710)
**IS therapy at IFI**			
Steroids	14	13	27
CSA or FK 506 *	8	6	14
**Number of organ involved**			
1	17	12	29
2	3	4	7
≥ 3	2	2	4
**Type of organ involvement**			
Lung	22	17	39
CNS	3	1	4
Skin	1	2	3
URA	2	0	2
Heart	0	2	2
Other	1	6	7
**Surgery**	7	7	14
Site			
Lung	6	5	11
other	1	2	3
Time from diagnosis of IA to surgery			
Median (days)	27	20	26
range	(16–218)	(0–81)	(0–218)

Group A comprises patients given caspofungin-based combination therapy starting within 7 days from diagnosis of IA; Group B consists of patients given caspofungin-based combination therapy starting after 7 days from diagnosis of IA.

IS, immunosuppressive drugs; IFI, invasive fungal infection; CNS, central nervous system; URA, upper respiratory airways; *with or without other IS drugs; CSA, cyclosporine-A, FK 506, tacrolimus.

Severe neutropenia was present at diagnosis of IA in 31 patients (78%) and lasted for a median time of 29 days, range 2–251. The median lymphocyte count at time of diagnosis of IA was 170 × 10^9^/L, range 0–5,710.

In 27 (68%) episodes, the patient was on steroid therapy at diagnosis of IA, whilst in 14 (35%) episodes the patient was receiving either cyclosporine-A or tacrolimus.

The lungs were the most frequent organ affected, pulmonary IA being involved in 39 (98%) out of the 40 children enrolled in this analysis. Most patients, 29 (73%), had involvement of a single site: lung, 28; skin, 1; whilst 11 patients (27%) had 2 or more organs involved. In this last group, central nervous system involvement was present in 4 patients.

Thirty-three of 40 patients (82.5%) were on antifungal treatment before starting the combination therapy, as follows: 13 patients were on prophylaxis with fluconazole (12 patients) or itraconazole (1 patient) for a median time of 28 days (range 10–50); 6 patients were on liposomal amphotericin-B as empiric treatment of fever and neutropenia for a median time of 10 days (range 3–43); and 14 patients were on liposomal amphotericin-B (10 patients) or caspofungin (3 patients) or voriconazole (1 patient) as first-line treatment of IA for a median time of 8 days (range 3–56). The reasons for switching to combination antifungal therapy in the patient on antifungal therapy were progression of disease or failure to improve clinically or radiographically. The remaining 7 patients were started on combination therapy as first-line treatment of IA.

Fourteen patients (35%) underwent elective surgical resection of the fungal lesions, from lungs 11 (27.5%) or from other organs 3 (7.5%), after a median time of 26 days (range 0–218 days) from diagnosis of IA.

*Response to therapy and survival*: the median duration of any combination of antifungal therapy was 29 days (range 3–382 days). Considering the 36 patients who received at least seven days of combination therapy, 18 patients (50%) received the combination of caspofungin and liposomal amphotericin B for a median time of 26 days (range 7–90 days), 9 patients (25%) received the combination of caspofungin and voriconazole for a median of 38 days (range 12–94 days) and the remaining 9 patients (25%) received sequentially both combinations, for a median of 19 days (range 7–84 days). The dosages used were as follows: caspofungin, 70 mg/m^2 ^the first day, followed by 50 mg/m^2^/day; voriconazole, 2 × 6 mg/kg the first day, followed by 2 × 4 mg/kg/day; and liposomal amphotericin B, 3–5 mg/kg/day. No difference was found in terms of outcome at 100 days from diagnosis of IA in these 3 groups of patients, a favorable response being observed in 39%, 56% and 56%, respectively, (p NS).

**Figure 1:**
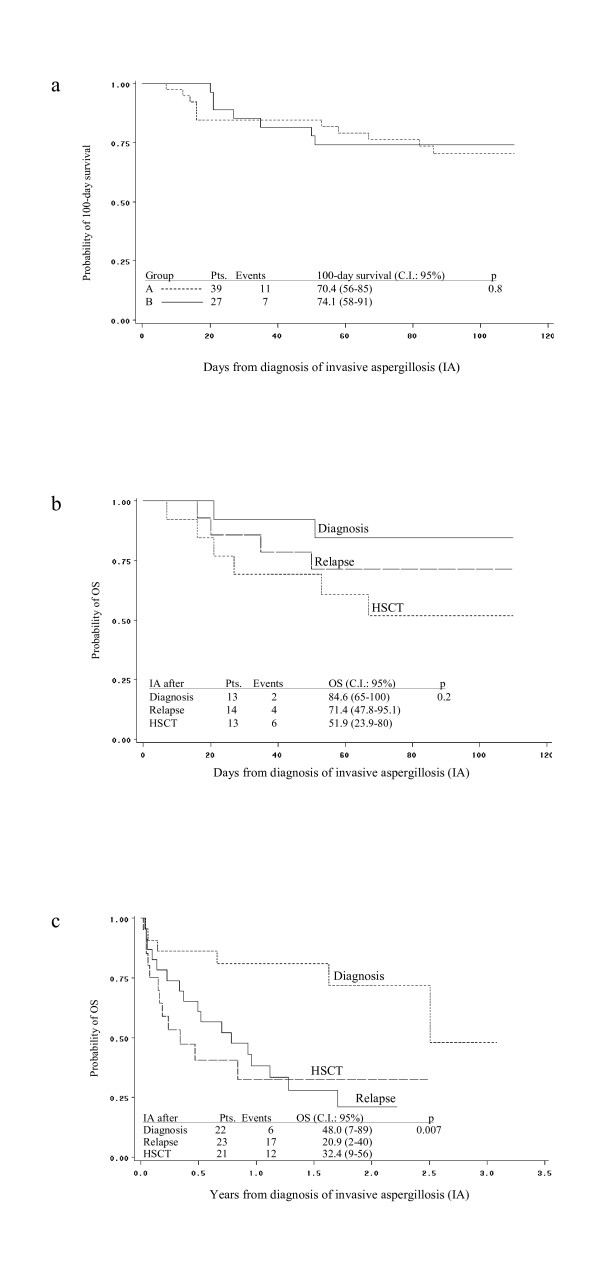
a) No difference was found in the 100-day survival between the patients of group A, who were started on a caspofungin-based combination therapy within < 7 days from diagnosis of IA vs the patients of group B, who were started on a caspofungin-based combination therapy after 7 days from diagnosis of IA. b) and c) Kaplan-Meier estimate of 100-day survival and OS according to the timing of diagnosis of IA: IA after diagnosis of underlying disease vs IA after HSCT vs IA after relapse.

At 100–days from diagnosis of IA, complete response was observed in 12 patients (30%), partial response in 9 patients (22.5%), stable response in 4 patients (10%) and failure in 2 (5%) patients. Noteworthy, 2 of 4 patients with CNS involvement had significant clinical improvement and were classified as partial responder. Overall, a favourable response was documented in 21 of 40 patients (53%). One-hundred-day survival was 70%, confidence interval (C.I.) being 55–84. Figure 1a shows that no difference was observed for the 100-day survival according to the timing of adoption of a caspofungin-based combination therapy: group A, 70.4%, C.I. 56–85; vs group B, 74.1%, C.I. 58–91, p 0.8. Moreover, 100-day survival resulted different, though not significant, according to the timing of diagnosis of IA: 84.6% (C.I. 65–100) for IA occurring after diagnosis of underlying disease; 71.4% (47.8–95.1) for IA occurring after relapse of underlying disease; and 51.9% (C.I. 23.9–80) for IA occurring after HSCT, p 0.2 (figure 1b).

After a median follow-up of 0.7 years (range 100 days – 3.1 years), 20 patients (50%) are alive. The probability of OS for the whole group of patients was 44%, C.I. 27–61. Figure 1c shows that OS probability was significantly better in patients who developed IA after diagnosis of underlying disease, 48% (C.I. 7–89), as compared with those experiencing IA either after relapse, 20.9%, (C.I. 2–40), or after HSCT, 32.4% (C.I. 9–56), respectively, p 0.007.

Sixteen of 20 patients (80%) who died had active aspergillosis before death on the basis of clinical, radiological and microbiological investigations; 8 of these 16 patients (50%) were either not in remission of their underlying disease or had clinical and/or hematological signs of progression of their primary disease. The other causes of death were not related to IA but to progression of the primary malignant disease, 1 (5%), graft-versus-host disease, 2 (10%) and septic shock, 1 (5%).

*Analysis of factors predictive of favorable response, 100-survival and OS: *table 3 shows the results of analysis on potentially prognostic factors. No factor was found to predict a favourable response in univariate and multivariate analysis.

**Table 3:** Univariate and multivariate analysis of the factors associated with favorable response to antifungal therapy, 100-day survival and overall survival rate

**Factors**	Favorable response rate	Univ P	Multiv P	100-day-survival rate	Univ P	Multiv P	OS rate	Univar P	Multiv P
Gender									
M	11/21	1		15/21	0.8		9/21	0.3	
F	10/19			13/19			11/19		
Age (years) at IA									
< the median	10/20	0.8		15/20	0.5		12/20	0.2	
≥ the median	11/20			13/20			8/20		
IA after:									
HSCT/Relapse	12/27	0.1		17/27	0.3		10/27	0.02	0.048
Diagnosis	9/13			11/13			10/13		
ANC < 0.5 × 10^9^/l									
yes	16/31	1		21/31	0.7		14/31	0.5	
no	5/9			7/9			6/9		
Number of organ affected:									
Single organ	14/29	0.4		20/29	1		12/29	0.08	
Multiple organs	7/11			8/11			8/11		
Type of IA									
Probable IA	8/20	0.1		11/20	0.04	-	6/20	0.01	
Documented IA	13/20			17/20			14/20		
Surgical treatment						-			
Yes	9/14	0.3		13/14	0.03		10/14	0.047	
No	12/26			15/26			10/26		
Timing of caspofungin-based combination therapy									
< 7 days from IA	13/22	0.4		17/22	0.3		10/22	0.5	
≥ 7 days from IA	8/18			11/18			10/18		
Combination therapy as:									
Primary or	12/20	0.3		14/20	1		10/20	1	
Salvage therapy	9/20			14/20			10/20		
Favorable response									
yes	NE	NE	NE	21/21	< 0.001	-	16/21	< 0.001	0.003
no				7/19			4/19		

Univ.: univariate analysis; Multiv.: multivariate analysis; OS: overall survival; M: male; F: female; IA: invasive aspergillosis; ANC: absolute neutrophil count; NE.: not evaluated;

Factors significantly associated in univariate analysis with a better 100-day survival and OS were type of IA, surgical treatment, and the achievement of a favourable response rate.

In multivariate analysis, no factor was predictive for 100-day survival whilst the favorable response to antifungal therapy and the occurrence of IA after diagnosis of underlying disease vs. after HSCT or relapse resulted significantly associated to better OS, p 0.03 and p 0.04, respectively.

*Safety and toxicity*: the antifungal combination therapy was generally well tolerated and no severe renal or liver impairment (grade II-IV WHO toxicity) attributable to the antifungal drugs was observed. Two patients were withdrawn whilst on voriconazole, for bradi-arrhythmia, 1; and diarrhea and bone pain, 1, respectively. One patient developed a transient skin rash while on caspofungin therapy; this side effect was controlled with symptomatic drugs and did not require withdrawal of caspofungin.

## Discussion

Despite recent advances in diagnosis and treatment, IA still represent an important cause of mortality in immune-compromised hosts. [2-5] In this last few years, a new strategy of antifungal therapy, using a combination of antifungal drugs, has been an important subject of investigation, and as a whole, the available experimental and clinical results suggest that combination antifungal therapy may improve patient outcome. [30] The recent introduction of new molecules with broad spectrum of activity, low toxicity, and novel mechanisms of action such echinocandin is certainly favoring this strategy. There are several arguments that justify the strategy of combining antifungal drugs to optimize therapy such as the *in vitro *data showing a potential for a synergistic effect, broader spectrum of activity and a decreased risk of emergence of resistant strains; and the absence of a negative or harmful effect when an azole is combined with a polyene or an echinocandin in animal models of IA. [30-34]

Recent reports have suggested that combination therapy with the new antifungal drugs may be more effective that antifungal mono-therapy, raising the hope that new standards for the treatment of invasive mycoses may become available in the near future. [10-15]

Our study represents the largest survey on the use of combination antifungal therapy for IA in children and adolescents. The first observation deriving from this study is that the use of combination therapy is definitely increasing compared with past practice. According to published data, only 249 cases of combination antifungal therapy were reported among 6,281 total cases of IA diagnosed in the period from 1966 to 2001. [30] In contrast, we collected 40 episodes of combination therapy in the 36-month duration of this study (1.1 episodes/month). A recent prospective multicenter surveillance survey from 2001 to 2002 among the AIEOP centers showed that 49% of episodes of fungemia, or proven, probable and possible mycoses have been treated with combination therapy [19].

Differently from other authors [10,11], we excluded from analysis patients with a lower certainty of IA, namely children fulfilling the criteria of possible IA, as we reasoned that these cases should be considered not sufficiently reliable to assess the efficacy of an antifungal drug. As a whole, only 17 patients with diagnosis of possible IA were treated with combination therapy during the study period (data not shown).

Overall, the favorable response rate and 100-day survival were 53% and 70% respectively, which are noteworthy if one considers our high-risk population where 31 patients (78%) had severe neutropenia; 27 patients (67.5%) had IA after relapse of underlying disease or after HSCT; and 20 patients (50%) were refractoriness to empiric or first-line antifungal therapy. Despite this, we obtained a response rate and 100-survival superimposable to that observed in two recent randomized studies where voriconazole and liposomal amphotericin-B (3 mg/kg) were used as first-line treatment for IA i.e. the favorable response rate was 52.8% and 50%; and the 12-week survival 70.8% and 72%, respectively. [5,35] Moreover, combination antifungal therapy was associated to a superior successful outcome than previous studies with newer agents used as salvage mono-therapy: amphotericin B-lipid complex, 42% [36]; voriconazole 39% [29]; caspofungin, 45%–44%. [37,38]*Walsh et al*. reported a favorable response rate of 33% and 38% in children with either hematological malignancy or given HSCT who received voriconazole as rescue therapy for IA.[39] A recent prospective, open-label, non-comparative study on 53 adults reported the results of caspofungin given with other antifungals as salvage therapy for IA refractory (87%) or intolerant (13%) to first-line antifungal therapy. Favorable response at the end of combination therapy was 55% whilst 12-week favorable response and survival rate was 49% and 55%, respectively [40]

The overall survival of 44% of our patients compared well with that observed retrospectively by Abbasi et al. in 66 pediatric cancer patients cancer where 1-year mortality was 85%; moreover, in that study lung involvement was associated with poorer outcome.[41]

In patients given allogeneic HSCT, IA is still associated to lower response to antifungal drugs and survival. This is confirmed by recent study on adult HSCT patients, who had been diagnosed with proven or probable IA in 2002, that showed a discouraging 4-month survival of 34%. [42]

We found a response rate and a 100-day survival of 44% and 63% in patient with IA after HSCT or relapse that compared well with the results observed for the same subgroup of patients in the studies with voriconazole or caspofungin as salvage mono-therapy [29,37]. Our results are in line with those of Marr et al. who showed that combination therapy with caspofungin and voriconazole in HSCT patients gave a better 3-month survival than that observed in a historical control group treated with voriconazole alone.[13] However, the retrospective design of this study did not allow to ascertain clearly if the combination caspofungin-based combination therapy was significantly better than any other monotherapy without caspofungin.

Neither the type of combination antifungal therapy nor the early introduction of caspofungin-based combination therapy had a significant effect on the response rate and 100-day survival. In the multivariate analysis, the only factors that resulted significantly associated to better overall survival were the achievement of a favourable response to antifungal treatment and the occurrence of IA after the diagnosis of underlying disease.

## Conclusion

This study has shown that the combination of caspofungin with other antifungal drugs is effective and well tolerated also in pediatric patients with IA. Despite the inclusion of patients with high-risk characteristics of poor outcome, favorable response and 100-day survival were not inferior to those reported for antifungal mono-therapy with new antifungal drugs used as first-line or rescue treatment for IA. The potential benefit of combination antifungal therapy over mono-therapy needs to be investigated by prospective controlled studies.

## Competing interests

EC received grant for lectures by Pfizer, Gilead and Merck. SC received grant for lecture by Gilead and Merck. All the other authors declare no conflict of, and/or competing interests.

## Authors' contributions

SC conceived of the study and drafted the manuscript; MG, EC, MRR and FL participated in its design and coordination and contributed to draft the manuscript; MS, BB, EG, FT, DC collected the data; SC and GT performed the statistical analysis. All authors read and approved the final manuscript.

## Pre-publication history

The pre-publication history for this paper can be accessed here:


